# Should I Lay or Should I Grow: Photoperiodic Versus Metabolic Cues in Chickens

**DOI:** 10.3389/fphys.2020.00707

**Published:** 2020-06-26

**Authors:** Charlene Hanlon, Ramesh Ramachandran, Martin J. Zuidhof, Grégoy Y. Bédécarrats

**Affiliations:** ^1^Department of Animal Biosciences, University of Guelph, Guelph, ON, Canada; ^2^Center for Reproductive Biology and Health, Department of Animal Science, Pennsylvania State University, University Park, PA, United States; ^3^Department of Agricultural, Food and Nutritional Science, University of Alberta, Edmonton, AB, Canada

**Keywords:** sexual maturation, laying hen, metabolism, photoreception, HPG axis

## Abstract

While photoperiod has been generally accepted as the primary if not the exclusive cue to stimulate reproduction in photoperiodic breeders such as the laying hen, current knowledge suggests that metabolism, and/or body composition can also play an influential role to control the hypothalamic-pituitary gonadal (HPG)-axis. This review thus intends to first describe how photoperiodic and metabolic cues can impact the HPG axis, then explore and propose potential common pathways and mechanisms through which both cues could be integrated. Photostimulation refers to a perceived increase in day-length resulting in the stimulation of the HPG. While photoreceptors are present in the retina of the eye and the pineal gland, it is the deep brain photoreceptors (DBPs) located in the hypothalamus that have been identified as the potential mediators of photostimulation, including melanopsin (OPN4), neuropsin (OPN5), and vertebrate-ancient opsin (VA-Opsin). Here, we present the current state of knowledge surrounding these DBPs, along with their individual and relative importance and, their possible downstream mechanisms of action to initiate the activation of the HPG axis. On the metabolic side, specific attention is placed on the hypothalamic integration of appetite control with the stimulatory (Gonadotropin Releasing Hormone; GnRH) and inhibitory (Gonadotropin Inhibitory Hormone; GnIH) neuropeptides involved in the control of the HPG axis. Specifically, the impact of orexigenic peptides agouti-related peptide (AgRP), and neuropeptide Y (NPY), as well as the anorexigenic peptides pro-opiomelanocortin (POMC), and cocaine-and amphetamine regulated transcript (CART) is reviewed. Furthermore, beyond hypothalamic control, several metabolic factors involved in the control of body weight and composition are also presented as possible modulators of reproduction at all three levels of the HPG axis. These include peroxisome proliferator-activated receptor gamma (PPAR-γ) for its impact in liver metabolism during the switch from growth to reproduction, adiponectin as a potential modulator of ovarian development and follicular maturation, as well as growth hormone (GH), and leptin (LEP).

## Introduction

Decades of genetic selection along with significant improvements in environmental conditions and nutrition have allowed modern commercial chickens to become exceedingly efficient. However, as traits associated with growth and reproduction are negatively correlated (Siegel and Dunnington, 2017), divergent breeding objectives have been established for broiler and layer chickens. While breeding programs for layers have been instrumental in improving production by advancing sexual maturation, reducing the time of egg formation and improving peak of lay, limited emphasis was put on the underlying physiological processes, thus pushing the boundaries closer to the hen’s biological limit (van Sambeek, 2010). With the rising demand for sustainable egg production, breeding companies have more recently focused on extending the laying period to achieve 500 eggs per hen at 100 weeks of age (van Sambeek, 2010; Bain et al., 2016). Physiologically, this will require precise co-ordination of several systems involved not only in the control of the reproductive axis, but also metabolism and nutrient partitioning. However, to date, most research models describing the control of the hypothalamic-pituitary gonadal (HPG) axis have largely focused on the impact of environmental cues such as photoperiod (Bédécarrats, 2015), rather than incorporating the impact of growth and metabolic status. Recent evidence suggests that modern commercial laying hens do not exclusively rely on photostimulation to initiate sexual maturation, as egg production may commence at an earlier age, prior to an increase in photoperiod (Baxter and Bédécarrats, 2019).

Conversely, broiler chickens have been intensively selected for increased growth rate and feed efficiency (Zuidhof et al., 2014). As a result, broiler breeders, the parent stock of broilers, carry the genetics for rapid growth, while displaying poor reproductive capacity in comparison to layers. Specifically, when fed *ad-libitum*, breeders tend to rapidly become overweight due to a lack of appetite control. In turn, this results in compromised health, along with impaired reproduction. Thus, pullets are typically reared under feed restriction programs (Decuypere et al., 2010). Although the impact of body weight on reproductive fitness has been studied in broiler breeders (Eitan et al., 2014; van der Klein et al., 2018) and migratory birds (Davies and Deviche, 2014), this aspect has been largely overlooked in laying hens. While it is accepted that laying hens need to achieve a mature body weight prior to sexual maturation (Brody et al., 1984; Dunnington and Siegel, 1984; Zelenka et al., 1987), little is known about the physiological conditions and body composition underlying this suggested threshold.

As more evidence of convergence between hormones influencing both metabolic control and reproductive processes emerges, it is imperative to further study and describe these interactions. Thus, this review aims to summarize the current knowledge on the control of sexual maturation in chickens, with a specific emphasis on the integration of photoperiodic cues while presenting evidence of possible interactions with factors involved in metabolic control.

## Photoperiodic Control of Reproduction

### Overview of the Effects of Photoperiod on the Reproductive Axis

Early studies conducted in wild birds showed that increased day length during spring coincided with increases in gonadal weight and size (Homma et al., 1994) and the initiation of reproduction and breeding (Whetham, 1933; Byerly and Moore, 1941; Sharp, 1993). This concept of reproductive modifications in response to changing seasonal day length served as starting point for the development of lighting programs under managed environments. Since then, the integration of photoperiodic signals on the activation and function of the HPG axis has been fairly well characterized and reviewed elsewhere (Sharp, 2005; Bédécarrats et al., 2009; Bédécarrats, 2015; Bedecarrats et al., 2016). This axis is primarily responsible for providing a cohesive signal, through the coordinated synthesis and secretion of hormones to effectively initiate or terminate the reproductive cycle. The hypothalamus, which acts as a neuroendocrine control center, is responsible for secreting stimulatory neuropeptides, gonadotropin-releasing hormones (GnRH-I and GnRH-II), along with an inhibitory neuropeptide, gonadotropin-inhibitory hormone (GnIH; Matsuo et al., 1971; Tsutsui et al., 2000). In turn, these neuropeptides regulate the synthesis and release of gonadotropins which then lead to the activation of the ovary, allowing for the initiation of lay. In vertebrates, light, hence photoperiod, is detected by photoreceptors and transduced into nervous and endocrine signals (Ebrey and Koutalos, 2001). In avian species, these photoreceptors are present in the eye as visual photoreceptors on the retina, as well as in the pineal gland and the hypothalamus, as extra-retinal photoreceptors (Kumar et al., 2004) to coordinate photoperiodic responses.

Although the role of the pineal gland and its associated melatonin (MEL) production has not been directly linked to the ability of the photoperiodic response to trigger reproduction (Juss et al., 1993; Bentley et al., 2013; Kang and Kuenzel, 2015), MEL released by the pineal gland and the retina during the scotophase stimulates the expression of GnIH by the hypothalamus (Ubuka et al., 2005). During the pullet growing stage, when the chicken is sexually immature, photoperiod is maintained below 10 h of light, resulting in elevated levels of MEL, thus maintaining inhibition via GnIH. In addition to directly supressing GnRH production (Bentley et al., 2003, 2008) and release (Tsutsui et al., 2000), GnIH also supresses the hormonal response from the anterior pituitary by binding to its gonadotropin-inhibitory hormone receptor (GnIH-R) and preventing the secretion of gonadotropins, luteinizing hormone (LH), and follicle-stimulating hormone (FSH; Ciccone et al., 2004; Ikemoto and Park, 2005; Ubuka et al., 2006). At the time of photostimulation, reduced scotophase results in lower MEL synthesis, thus lifting the inhibition on the HPG axis by GnIH (Ikemoto and Park, 2005; Maddineni et al., 2008). Suppression of MEL production occurs through the stimulation of the pineal gland specific photoreceptor, pinopsin (Okano et al., 1994; Holthues et al., 2005). The resulting down-regulation of GnIH removes the suppression of GnRH and gonadotropes, allowing the pituitary to synthesize and release LH and FSH (Ikemoto and Park, 2005). Upon photostimulation, a longer day length will not only reduce the production of MEL, but also stimulate deep brain photoreceptors (DBPs) to trigger a greater synthesis and release of GnRH from the hypothalamus. Although two different isoforms of chicken GnRH have been characterized, GnRH-I and GnRH-II, which both bind to the GnRH receptors (cGnRH-RI and cGnRH-RIII), it is now well accepted that GnRH-I is the neuropeptide released in the median eminence (ME) to stimulate pituitary gonadotropes by binding to cGnRH-RIII, the predominant receptor present in the pituitary gland (Shimizu and Bedecarrats, 2006; Joseph et al., 2009). Interestingly, it was shown that the pituitary ratio of GnIH-R to cGnRH-RIII switches at the time of sexual maturation (Shimizu and Bédécarrats, 2010), thus also shifting the sensitivity of the pituitary from inhibitory to stimulatory.

As GnRH increases, circulating levels of LH and FSH increase (Etches, 1996). Both glycoproteins are composed of 2 subunits including a common alpha subunit, along with a unique beta subunit responsible for their specific actions (Burke et al., 1979). While the major role of LH is the induction of ovulation through an increase in progesterone and testosterone production by the ovary of the mature hen (Shahabi et al., 1975), during the earlier stages of sexual maturation and follicular development, LH stimulates steroidogenesis of various sex hormones, such as androgens, estrogens, and progestins by the follicles and ovarian cortex (Robinson et al., 1988, 2003). On the other hand, the primary role of FSH during the follicular maturation process includes granulosa cell differentiation and the stimulation of steroidogenesis, as the pre-hierarchal follicles acquire FSH responsiveness (Johnson and Bridgham, 2001; Johnson and Woods, 2009a). In culture, administration of FSH demonstrated an increase in the production of cAMP in the smaller pre-ovulatory follicles, while this effect of FSH was reduced as these follicles increase in size and become responsive to LH (Calvo and Bahr, 1983). Meanwhile, daily exogenous doses in laying hens were able to increase the number of white follicles, small yellow follicles, and preovulatory follicles, demonstrating the critical role of FSH in all stages of follicular development (Palmer and Bahr, 1992). Thereby, both gonadotropins influence the steroidogenic capacity of the ovary, enabling the production and secretion of estradiol (E_2_) and progesterone (P_4_; Shahabi et al., 1975; Shodono et al., 1975). At the time of sexual maturation, the ovary contains thousands of viable small white follicles (SWF) embedded in the highly vascularized stroma of the ovary (Johnson and Woods, 2009b), and these follicles are responsible for the production of circulating E_2_ (Robinson and Etches, 1986). The rise in E_2_ following photostimulation results in amplified protein synthesis to initiate maturation of the oviduct (Muller et al., 1970), with this demand further increasing prior to the onset of lay when elevated concentrations of E_2_ and P_4_ are present (Shahabi et al., 1975; Shodono et al., 1975). Due to the oviparous nature of this species, during maturation E_2_ targets the liver for the synthesis of yolk proteins deposited into follicles, the skeletal frame and digestive system to coordinate calcium mobilization for shell formation, and the oviduct for the coordination between egg formation and ovulation (Dacke et al., 1993; Walzem et al., 1999).

### Photoreception and Photoreceptors

#### Retinal Photoreceptors

The retina of the eye, through the capture and absorption of photons, is the primary source of photic information by receiving and transmitting images from the external environment to the brain while also contributing in part to the entrainment of the circadian rhythm (Underwood et al., 1984). The retina has 3 types of photoreceptor cells classified as rods, cones and double cones (Perry, 1995). Rod cells are primarily utilized during periods of low illumination, as they are highly sensitive to light, yet they do not detect colour due to the single spectral class (Bowmaker and Knowles, 1977; Yau, 1994; Hart, 2001). Conversely, cone cells can be used at much higher levels of brightness and to determine variations in colour, with avian species being tetrachromatic, meaning they are able to visualize peaks within violet (415 nm), blue (455 nm), green (508 nm), and red (571 nm) wavelengths (Yoshizawa, 1992; Perry, 1995; Prescott and Wathes, 1999; Hart, 2001). Initially, retinal photoreceptors were believed to be the only types of photoreceptors, as an early study on migrating junco showed that providing supplemental light to artificially create long days (LDs) resulted in hens laying eggs in the middle of the winter season (Rowan, 1931). However, it was later determined that blind laying hens have a similar rate of egg production when compared to their sighted counterparts, regardless of the presence of retinal photoreceptors (Siopes and Wilson, 1980). This study was one of the first to suggest that the eye is a non-essential component of a hens’ neuroendocrine reflex to light, indicating it may not be necessary for the photostimulatory response. Since then, further studies have suggested that in fact, input from the retinal photoreceptors may delay sexual maturation, as blind hens and roosters were shown to mature more rapidly than their sighted counterparts (Siopes and Wilson, 1980; Perttula and Bédécarrats, 2012; Baxter et al., 2014).

#### Extra-Retinal Photoreceptors

With retinal photoreceptors playing no significant role in mediating the response to photostimulation, transduction of light photons into biological signals is believed to be performed by extra-retinal reception. One of the first studies to show the significance of extra-retinal photoreceptors was conducted by Benoit in 1935 (Benoit, 1935a,b), showing that although sighted and blind ducks displayed similar gonadal growth rates and patterns, when a black cap was placed over the head to prevent deep brain penetration of light, the gonadal response was inhibited. This implied that the avian skull is permeable to light and that light could scatter through and be absorbed by overlapping tissues in order to stimulate encephalic receptors (Foster and Follett, 1985). Thus, emphasis will be put on deep brain photoreception, as the role of the pineal gland and its associated MEL production were discussed in the previous section.

Any photoreceptive molecule outside of the retina is referred to as an extra-retinal photoreceptor. In the avian brain, 4 regions have been proposed to house DBPs; the lateral septal region (LSO), the paraventricular nucleus (PVN), the premammillary nucleus (PMM), and the paraventricular organ (PVO; Kuenzel et al., 2015). Four criteria have also been utilized to determine if new candidate photopigments can be classified as a DBP, including (1) Explicit expression in the photosensitive region of the brain; (2) Physiological capability of the molecule to signal light as an opsin/vitamin A-based photopigment; (3) Appropriate maximum spectral absorption, predicted to be ∼492 nm, and (4) Corresponding to the maximum photon capture and spectrum of available light within the hypothalamus (Foster and Helfrich-Förster, 2001; Davies et al., 2012). From there, working models suggest that the components of the avian photoperiodic response are confined to the medio-basal hypothalamus (MBH; Benoit, 1935a,b; Menaker, 1968). To date, it has been proposed that the DBPs involved in priming the reproductive axis include the vertebrate ancient (VA) opsin (a member of the opsin 1 family; OPN1; Foster et al., 1985, 1994; Halford et al., 2009; Davies et al., 2012), melanopsin (OPN4; Chaurasia et al., 2005; Kang et al., 2010), and neuropsin (OPN5; Nakane and Yoshimura, 2010; Ohuchi et al., 2012).

### Candidate DBPs for Photo-Induced Sexual Maturation

Since the first discovery of DBPs, new opsin families have been identified in the hypothalamus. The recent annotation of opsin sequences within the chicken genome has allowed for the identification of five opsin family categories under which all opsins can be classified, including OPN1, OPN3, OPN4, OPN5, and retinal G-protein coupled receptors (RGR; Table 1). The family of OPN1 includes VA-opsin, expressed in the hypothalamus and pineal gland, as well as rhodopsin, expressed in the retina and pineal gland (Foster et al., 1985, 1994; Halford et al., 2009; Davies et al., 2012). Conversely, members from the OPN3 family consist of teleost multiple tissue opsins (TMTs) found in the cerebellum, retina and the PVN of the hypothalamus, and encephalopsins found in the cerebellum as well as the thalamic nuclei (Kato et al., 2016), suggesting this family does not play a role in reproductive control due to its localization outside the light-sensitive regions associated with reproduction. Similarly, with RGR expressed in the retina and pineal gland rather than the brain, evidence supports a role in circadian rhythm and vision rather than reproduction (Díaz et al., 2017). Meanwhile, two additional candidates, OPN4 and OPN5 were identified within various photosensitive regions throughout the hypothalamus (Chaurasia et al., 2005; Kang et al., 2010; Nakane and Yoshimura, 2010; Ohuchi et al., 2012), with OPN4 also expressed in the pineal gland (Chaurasia et al., 2005; Kang et al., 2010), and the retina (Tomonari et al., 2005). Thus, based on location, OPN1, OPN4, and OPN5 appear to be the best candidates to act as mediators of photoperiod on reproduction and are further discussed below.

**TABLE 1:** Summary of the candidates for deep brain photoreception.

	**Opsins**	**Wavelengths**		**DBP**			
**Family**	**consolidated**	**(nm)**	**Expression**	**criteria**			
				**1**	**2**	**3**	**4**
OPN1	Vertebrate ancient (VA) Opsins	450–520	Pineal gland Hypothalamus	✓	✓	✓	✓
	Rhodopsin	480–495	Pineal gland Skin Retina	x	✓	✓	✓
	Pinopsins	480–540	Pineal gland	x	✓	✓	✓
OPN3	Teleost multiple tissue (TMT) opsins	450–470	Cerebellum Retina Paraventricular nucleus	x	✓	x	x
	Encephalopsins		Cerebellum Thalamic nuclei	x	✓	x	x
OPN4	Melanopsins	410–480	Hypothalamus Pineal gland Retina	✓	✓	x	x
OPN5	Neuropsin	350–470	Hypothalamus	✓	✓	x	x
RGR	Retinal G protein-coupled receptors	470–490	Retina	x	✓	x	x
	Peropsins		Pineal gland	x	✓	x	x

*The five families of opsins broken down into their components, along with their associated wavelength of spectral absorption (nm), the signaling pathway they utilized and where they are expressed in the chicken. Wavelengths indicated in BOLD are within the 492 nm maximum spectral absorption hypothesized to be associated with reproduction. The deep brain photoreceptor (DBP) criteria refer to (1) Explicit expression in photosensitive regions of the brain; (2) Physiological capability of the molecule to signal light as an opsin/vitamin A-based photopigment; (3) Appropriate maximum spectral absorption, predicted to be ∼492 nm; and (4) Correspond to the maximum photon capture and spectrum of available light within the hypothalamus (Foster and Follett, 1985; Davies et al., 2012).*

#### Melanopsin (OPN4)

Melanopsin is a photopigment often referred to as OPN4, due to its gene of origin (Hankins et al., 2008b). Originally isolated in the melanophores of *Xenopus* (Provencio et al., 1998), this protein was later found in the diencephalon, pineal gland, and retina of the chicken (Foster et al., 1987; Bailey and Cassone, 2005; Chaurasia et al., 2005; Tomonari et al., 2005; Kang et al., 2010) and, due to its role in non-image light detection, it was proposed as a candidate DBP impacting the HPG axis (Freedman et al., 1999; Peirson and Foster, 2006; Hankins et al., 2008b). At the time, two isoforms were identified in avian species including OPN4M (mammalian-like) and OPN4X (*xenopus*-like; Bellingham et al., 2006; Hankins et al., 2008a). In the turkey, OPN4X mRNA has been found in dopamine (DA)-expressing neurons of the brain, including in the PMM, as well as in MEL expressing neurons (Kang et al., 2010; Kosonsiriluk et al., 2012). The Dopamine-Melatonin (DA-MEL) neurons are activated upon interruption of the dark phase with various light periods from 30 min to 3 h when the birds are within the photoresponsive phase of reproduction, leading to decreased expression of OPN4X and MELergic activity, while DAergic activity is elevated (Kang et al., 2010). Increased DAergic activity has been implicated in GnRH-I and Vasoactive Intestinal Peptide (VIP) signaling (Bhatt et al., 2003; Chaiseha et al., 2003; Kang et al., 2006), possibly through the alteration of thyroid stimulating hormone (TSH), and type 2 deiodinase (DIO2) activity (Kang et al., 2010). While this evidence supports the role of melanopsin in the control of reproduction in the turkey hen, this connection has yet to be established in the domestic chicken. Although OPN4 is present in the brain of day-old chicks (Chaurasia et al., 2005), no expression has been found in the hypothalamus of maturing birds (Chaurasia et al., 2005; Hankins et al., 2008a). Furthermore, with an absorption spectrum between 410–480 nm, OPN4 fails to reach the predicted maximum spectral absorption of 492 nm for deep brain perception (Foster and Follett, 1985). Altogether, this indicates that while melanopsin may play a role in the photoperiodic response, it is unlikely to be the key opsin triggering the initiation of sexual maturation.

#### Neuropsin (OPN5)

Neuropsin is encoded by the OPN5 gene (Tarttelin et al., 2003), localized in the cerebrospinal fluid (CSF)-contacting neurons of the PVO within the MBH (Halford et al., 2009; Nakane and Yoshimura, 2010), meeting the location criteria outlined for a potential DBP. However, OPN5 is also expressed in the adrenal glands with a possible role in chemosensory reception (Ohuchi et al., 2012). Nonetheless, it has been the subject of a number of studies for its potential role in controlling reproduction. It was determined that OPN5 has two isoforms including an ultra-violet (UV) light-absorbing form that possesses a 11-*cis*-retinal with a maximum absorption at 360 nm, and a visible light-absorbing form altered by the addition of all-*trans*-retinal maximally absorbed at 474 nm (Yamashita et al., 2010). This indicates that this photopigment is bi-stable, signifying its ability to absorb two light spectra, and may interact with reproductive control in some capacity under either UV or visible light (Yamashita et al., 2010). While these molecules are both capable of signaling light within the hypothalamus, these absorption maxima fall short of the predicted wavelength requirements and, simply transitioning birds from short to LDs did not alter the expression of OPN5 (Yamashita et al., 2010; Stevenson and Ball, 2012). It has been proposed that light detected by OPN5-positive CSF-contacting neurons allows information to be transmitted to the pars tuberalis (PT) to induce TSH-β mRNA expression (Nakao et al., 2008), thereby suggesting that OPN5 plays a role in the activation of the HPG axis (Nakane et al., 2014). Interestingly, evidence that OPN5 is coupled to the G_i_ (inhibitory) subunit (Yamashita et al., 2010), along with OPN5 knockdown or gene silencing via small-interfering RNA (siRNA) disrupting the photoperiodic control of reproduction, has supported the theory that OPN5 could play an inhibitory role. One particular study used antisense sequences found to reduce the expression of OPN5 by 32% compared to the scrambled sequence and, birds demonstrated an elevation in TSH-β levels with the decline in OPN5 when photostimulated by LD under white light (Stevenson and Ball, 2012). A more recent study has demonstrated that knockdown of OPN5, in conjunction with pinealectomy and eye patches, suppressed the production of TSH-β traditionally stimulated by LD when housed under UV-lighting (Nakane et al., 2014). By utilizing UV-light, Nakane et al. (2014) were able to directly stimulate the OPN5 photoreceptors, explaining the opposing results of Stevenson and Ball (Stevenson and Ball, 2012), in which the photoreceptors non-responsive to UV stimulation had not been isolated. With an inhibitory impact in mind, future studies should explore a possible interaction between OPN5 and GnIH. Interestingly, it appears that expression of OPN5 is age-dependent, with expression increasing throughout maturity in male quail up to 16 weeks of age (woa), yet by 144 woa expression had decreased (Banerjee et al., 2018). Further studies in females would provide insight into these age-related changes in OPN5 and whether they correlate with sexual maturation and the dissipation of juvenile photorefractoriness.

#### Vertebrate Ancient (VA)-Opsin

Vertebrate Ancient-opsin, first identified in the Atlantic salmon (Soni and Foster, 1997; Soni et al., 1998), is a functional photopigment belonging to the OPN1 family. In the chicken, two isoforms, cVALong (cVAL), and cVAShort (cVAS), have been identified (Halford et al., 2009). With a spectral peak of 491 nm, perikarya localized in the MBH, and projections extending into the ME, VA-opsin satisfies all the proposed criteria for a DBP mediating photoperiodic response (Young, 1962; Hankins et al., 2008b; García-Fernández et al., 2015). Indeed, current working hypotheses suggest VA-opsin perikarya in the MBH are responsible for photoreception, with the projections sent to the posterior portion of the hypothalamus, through to the ME, allowing for interactions with the PT (García-Fernández et al., 2015). This would suggest that VA-opsin neurons may interact with pituitary thyrotropes to produce TSH, eliciting the response of thyroid hormones to activate the HPG axis, as described below. However, while these perikarya may be the primary site of photoreception, it is also possible that the fibers of VA-opsin neurons form a photosensitive net, responding to light directly within the ME rather than indirectly stimulating this region.

Recently, it was shown that VA-opsin is co-expressed with GnRH in perikarya present in the anterior and medial hypothalamus with projections to the ME (García-Fernández et al., 2015), corresponding to regions previously identified with GnRH (Foster et al., 1987; Dawson et al., 2001). While this suggests a direct link between VA-opsin photoreception and GnRH-I synthesis and release, the timing of GnRH-I release corresponds to the activation of the HPG axis (Ni et al., 2013), rather than anytime during the photosensitive period that precedes the trigger of photostimulation itself. Therefore, these discrepancies in timing signify that while this photoreceptor may interact with GnRH-I to activate sexual maturation, it is unlikely that VA-opsin is able to directly stimulate the HPG axis and its mode of action remains unknown. Similar to neuropsin, VA-Opsin mRNA, along with the number of ir-VA-Opsin cells, have been suggested to be elevated from the immature state through to the period of sexual maturation, from 6 to 16 woa in the male quail, with a decline observed at 144 woa (Banerjee et al., 2018). Furthermore, as VA-Opsin and GnRH-II perikarya have been identified to be expressed within similar regions, with projections extending to various additional regions of the brain outside of the ME (Sharp et al., 1990; García-Fernández et al., 2015), a possible relationship may exist and should be investigated. Interestingly, VA-Opsin also strongly co-localizes with arginine-vasotocin (AVT; García-Fernández et al., 2015), a system known to cause oviposition by triggering contractions of the shell gland in avian species (Koike et al., 1988), via an up-regulation of local prostaglandin production (Rzasa, 1978, 1984). Since AVT has also been linked to the stimulation of prolactin (PRL), adrenocorticotropic hormone (ACTH), and pro-opiomelanocortin (POMC; El Halawani et al., 1992; Wu et al., 2019), this raises the possibility that VA-opsin, in addition to contributing to the photo-induced activation of the HPG axis, may also contribute to the initiation of lay, as well as the control of oviposition timing via PRL (Harvey et al., 1979a). The implications of POMC along with its cleavage product ACTH on the neuroendocrine response of reproduction are discussed at length later in this review.

### Downstream Effects of Deep Brain Photoreception

While the exact characterization of hypothalamic photoreceptors remains elusive, the detection of light via photoreception and the cascade of succeeding events have been well established. It is known that longer daylength, integrated via a molecular clock contained within the MBH (Yasuo et al., 2003), will lead to the stimulation of thyrotrope cells in the PT of the pituitary to release TSH. TSH then acts on the specialized ependymal cells, referred to as tanycytes, contained within the third ventricle and believed to be critical for the induction of the HPG axis. Subsequent stimulation of these tanycytes will elicit an upregulation in the expression of DIO2 enzyme (Nakao et al., 2008). DIO2 is a thyroid hormone-activating enzyme responsible for the conversion of the prohormone, thyroxine (T_4_), into the bioactive form, triiodothyronine (T_3_; Bernal, 2002). It has been determined that DIO2 is directly induced through light stimulation during the photosensitive phases, however, this same elevation in expression is not observed when stimulation is provided outside of the photosensitive phase, meaning that DIO2 expression is upregulated under LD and downregulated under short day (SD; Yoshimura et al., 2003). At the same time, expression of a thyroid hormone-inhibiting enzyme, type 3 deiodinase or DIO3, was reported to act in an opposing fashion to DIO2 (Yasuo et al., 2005). The reciprocal relationship between these enzymes allows for a refined activity control of thyroid hormones within the MBH, occurring 18 h after dawn on the first day of photostimulation (Nakao et al., 2008). There is strong evidence suggesting TSH-β is a trigger for the expression of DIO2/DIO3. TSH-β is expressed in the PT 14 h after dawn, approximately 4 h prior to the release of DIO2/DIO3 (Nakao et al., 2008). This indicates that TSH under the influence of LD could be a key factor in the regulation of reproduction in birds (Yoshimura, 2013). Elevated levels of T_3_ in the MBH target thyroid hormone receptors in the ME (Yoshimura et al., 2003). GnRH nerve terminals, residing in the ME, will allow for the release of GnRH in response to these elevations, thereby activating the HPG axis (Hanon et al., 2008; Hazlerigg and Loudon, 2008; Nakao et al., 2008). This occurs as a result of morphological changes between GnRH nerve terminals and glial endfeet (Yamamura et al., 2004). Under SD, prior to the photostimulatory period, these GnRH nerve terminals are unable to contact the basal lamina as they are encased by the endfeet of glial processes. However, with the shift to LD, these nerve terminals are able to interact with the basal lamina, deemed critical as the neuropeptides must be secreted into the portal capillary system (Prevot et al., 1999; Yamamura et al., 2006). Additional studies have shown that local administration of T_3_ to the MBH has the ability to imitate these morphological changes to the GnRH nerve terminals, even under SD, outlining the importance of thyroid hormones to the reproductive process (Yamamura et al., 2006).

## Hypothalamic Integration of Appetite Control and Reproduction

### Appetite Control and the Melanocortin System

First and foremost, it is important to understand the integration of hypothalamic signals contributing to feed intake as it will ultimately impact body weight and composition of the hen. It is well established that the melanocortin system is responsible for monitoring energy status and controlling appetite. This occurs through the combined effects of POMC and cocaine and amphetamine-regulated transcript (CART) to downregulate hunger, as well as agouti-related peptide (AgRP), and neuropeptide Y (NPY) to upregulate feed intake. In addition, 5 melanocortin receptors have been identified, all of which are expressed in the avian brain (Takeuchi et al., 1996, 1998, 1996, 2000; Berghman et al., 1998; Takeuchi and Takahashi, 1998, 1999).

Initial studies showed that during periods of food deprivation or negative energy balance (Phillips-Singh et al., 2003; Higgins et al., 2010; Song et al., 2012), hens display elevated co-expression of orexigenic hormones, AgRP and NPY. Both peptides have been identified in the MBH of the ring dove (Strader and Buntin, 2003; Strader et al., 2003), as well as the infundibular nucleus (IN) in quail, which is the equivalent to the mammalian arcuate nucleus (ARC; Boswell et al., 2002), while NPY has been identified in the IN of the chicken (Kameda et al., 2001). Levels of AgRP have been reported to increase with the duration of food deprivation (Phillips-Singh et al., 2003; Higgins et al., 2010; Song et al., 2012), yet, strong evidence suggests these levels can be restored between 24 and 48 h following re-introduction to feed, depending upon the duration of deprivation (Harrold et al., 1999; Mizuno and Mobbs, 1999; Wilson and Bagnol, 1999; Higgins et al., 2010; Lei and Lixian, 2012; Song et al., 2012; Dunn et al., 2013; Fang et al., 2014). Levels of NPY mRNA were additionally found to elevate during periods of restricted feeding (Song et al., 2012) and, central injection of this peptide can stimulate feed intake in chickens (Kuenzel et al., 1987) and white crowned sparrows (Richardson et al., 1995). Thus through the coordination of NPY and AgRP (Hahn et al., 1998; Chen et al., 1999), the orexigenic portion of the melanocortin system is able to respond to declining energy status and hunger signals.

Conversely, gene expression of the anorexigenic hormones, POMC and CART, significantly decrease due to reduced food availability (Higgins et al., 2010). The co-expression of CART and POMC has not yet been published in chickens, yet CART neuronal cell bodies have been found in the IN of zebra finches (Singh et al., 2016). Although CART is able to decrease feed intake in *ad libitum* fed broilers and layers, it had no impact on feed restricted layers, while intracerebroventricular (ICV) injection did induce a dose-dependent decline in the feed intake of restricted broilers (Tachibana et al., 2003). Additionally, CART is able to partially inhibit NPY-induced feeding (Tachibana et al., 2003), but the mechanism of action is unknown. POMC is expressed in the IN of the hypothalamus, along with NPY and AgRP in quail (Boswell et al., 2002), as well as in chickens (Gerets et al., 2000). However, results have shown inconsistencies, as some studies have demonstrated that levels of POMC mRNA decrease after 7 days of restricted feeding in both layers and broilers (Hen et al., 2006), with others showing no changes in this anorexigenic peptide, demonstrating that AgRP mRNA is a much more reliable measurement (Dunn et al., 2013). Interestingly, while no differences in POMC mRNA were observed after 24 h of fasting, a significant decline occurred at 36 h (Ren et al., 2017), indicating that this hormone may not be the primary cause of the anorexigenic effects observed, but rather a result of the activated pathway. As a precursor polypeptide, POMC is cleaved into various key hormones, including ACTH, alpha-, beta-, and gamma-melanocyte stimulating hormone (α-MSH, β-MSH, and γ-MSH), and β-endorphin (Takeuchi et al., 1999). While α-MSH is involved in decreasing feed intake through the central nervous system by interacting with melanocortin receptor subtype 4 (MC4R; Mountjoy et al., 1994), ACTH is a key link between regulation of appetite and stress response via the hypothalamic-pituitary adrenal (HPA) axis (Aguilera, 1994), further decreasing feed intake when injected (Kawakami et al., 2000; Strader et al., 2003; Cline et al., 2008; Shipp et al., 2016). However, it has also been demonstrated that α-MSH can trigger the release of corticosterone (CORT) in a dose-dependent manner, supporting further integration of the HPA axis in appetite control (Tachibana et al., 2007). Yet, ostrich-β-endorphin has been reported to stimulate feed intake of pigeons immediately after ICV injection (Deviche and Schepers, 1984). The opposing role of this POMC cleavage product may offer insight into the aforementioned inconsistencies of the mRNA levels of the peptide during states of feed deprivation.

These melanocortins can elicit their response through any of the 5 receptor subtypes, however, unlike mammals, avian receptors possess a higher affinity for ACTH in comparison to that of α-MSH (Ling et al., 2004), pointing to a larger role for the stress response in appetite control. This alteration in avian species has been predicted to result from the absence of the intermediate lobe of the pituitary in the chicken, allowing ACTH to become the predominant melanocortin signal (Boswell and Takeuchi, 2005). Melanocortin receptors are able to stimulate various responses ranging from the regulation of energy expenditure, through receptor subtype 3 (MC3R), to food intake control, through MC4R. Due to the localization of MC3R and MC4R in the hypothalamus of the chicken, these subtypes have been heavily studied for their contributions to energy homeostasis (Ka et al., 2009; Higgins et al., 2010; Song et al., 2012; Yi et al., 2015). While α-MSH acts as an agonist of MC4R to inhibit feed intake in periods of satiety, AgRP has been found to antagonize the activity of ACTH and α-MSH on MC3R and MC4R (Zhang et al., 2017), demonstrating the ability of the melanocortin system to achieve energy homeostasis through competitive interactions with the receptors. However, each of these receptor subtypes are expressed in various tissues, ranging from the brain to the liver, playing a role in the integration of metabolic processes and appetite. Expressed in melanocytes, MC1R is primarily involved with α-MSH regulation of feather pigment (Teshigawara et al., 2001). Meanwhile, MC2R and MC3R are expressed in the adrenals, mediating the effects of ACTH on the HPA axis (Takeuchi et al., 1998; Takeuchi and Takahashi, 1999), and MC5R was the only receptor subtype present in the liver of the chicken (Ren et al., 2017). Additionally, NPY is known to act via its receptor subtypes, NPYR1 through NPYR5, which have been implicated in adipogenesis and early broiler growth (Resnyk et al., 2013; Shipp et al., 2016). Currently, NPYR1 has been hypothesized to be associated with most orexigenic activity elicited by NPY, according to studies conducted in mice (Gehlert, 1999). NPY1R and NPY5R mRNA have an elevated expression in the hypothalamus of low weight selected hens, compared to that of their high weight selected counterparts. However, these receptors were alternatively higher in adipose tissue of the high weight selected hens, compared to the low (Zhang et al., 2013). This differential receptor expression could implicate NPY in alternative pathways, diverting energy from fat storage to utilization, requiring further investigation in the sexual maturation of the laying hen.

Recently, melanocortin receptor accessory proteins (MRAPs) have been reported to support and alter the interactions of the receptors within the melanocortin system. In the chicken, MRAP is expressed in the brain, in addition to the adrenal gland, liver, spleen, stomach and lungs (Ren et al., 2017), while MRAP2 is expressed predominately in the brain (Asai et al., 2013). Currently, much of the work surrounding these accessory proteins has been completed in mammals, where MRAP2 has been linked to growth and metabolism (Cone, 2006; Asai et al., 2013; Sebag et al., 2013), interacting directly with MC4R in the brain to enhance cAMP production driven by the receptor in mice (Asai et al., 2013). Consequently, MC4R, linked to anorexigenic activity via ACTH, has also been found to act as an ACTH receptor in the presence of MRAP2 in zebrafish (Agulleiro et al., 2013). Additionally, in chickens it appears that while MRAP and MRAP2 have the ability to decrease MC4R and MC5R expression in the plasma membrane, they have no effect on the remaining MCRs (Ren et al., 2017).

**FIGURE 1:**
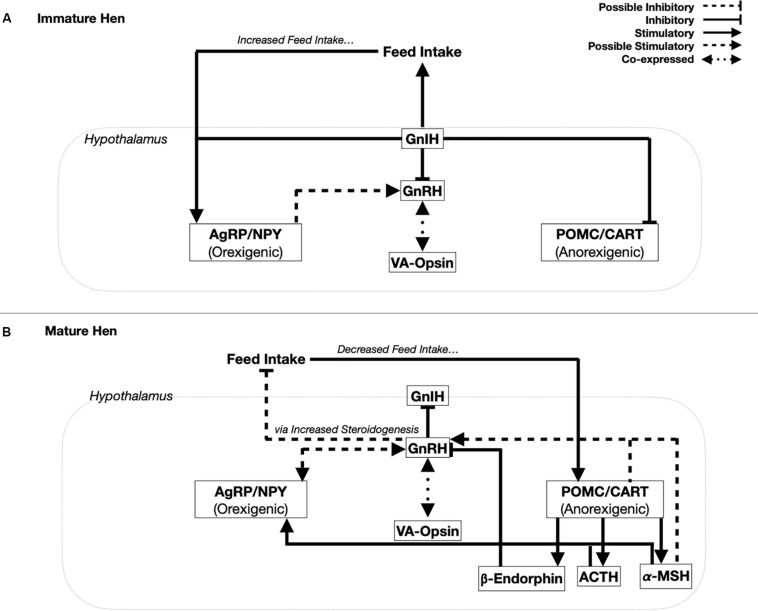
Proposed integration of the hypothalamic control of reproduction with the melanocortin system. While the hen is in an immature state **(A)**, Gonadotropin-inhibitory hormone (GnIH) is the primary neuropeptide released by the hypothalamus. GnIH simultaneously stimulates orexigenic peptides, agouti-related peptide (AgRP), and Neuropeptide Y (NPY), while inhibiting the anorexigenic peptides, pro-opiomelanocortin (POMC), and cocaine-and amphetamine regulated transcript (CART). Additionally, GnIH stimulates feed intake during a period of growth, also linked to AgRP and NPY upregulation. Recent evidence suggests that AgRP and NPY have the ability to stimulate gonadotropin-releasing hormone (GnRH), which may in turn activate the HPG axis. However, with the co-expression of vertebrate ancient (VA)-opsin and GnRH, this may be the primary site for mediating the effects of photoperiod. In the mature hen **(B)**, GnRH inhibits the production of GnIH and is proposed to decrease feed intake via an increase in steroidogenic activity. A decrease in feed intake stimulates POMC and CART activity, with POMC cleaved into 3 peptides, β-endorphin, adrenocorticotropin hormone (ACTH), and alpha-melanocortin-stimulating hormone (α-MSH). ACTH and α-MSH are able to stimulate AgRP and NPY. Meanwhile, CART, along with ACTH and α-MSH have been hypothesized to positively feedback on GnRH to stimulate the HPG axis. However, POMC cleavage may be critical in this control with β-endorphin established to inhibit GnRH release.

While the melanocortin system was initially believed to be the primary source of control involved in appetite regulation, involvement of these peptides in reproductive regulation has been proposed and will be discussed at length, as summarized in Figure 1. In addition, reproductive neuropeptides have been reported to play a role in the control of appetite and this integration will be considered.

### Integration Between the Melanocortin System and Reproduction

#### Effects of the Orexigenic System on Reproduction

NPY is one of the most potent orexigenic regulators of food intake (Hill et al., 2008; Pralong, 2010; Boswell, 2011), while also believed to control reproduction through its influence on ovulation (Dunn et al., 2004; Wu et al., 2007; Li et al., 2009). Predominantly, NPY is hypothesized to elicit its response through the stimulation of GnRH secretion (Contijoch et al., 1993), as NPY perikarya are located in the hypothalamus, with mRNA, peptide, and fibers located in the ME and fibers in the PVN, aligning with the location of GnRH-I and GnIH perikarya and fibres (Kuenzel, 2000; Singh et al., 2013). In addition, central injections of NPY can induce a premature LH surge in chickens (Contijoch et al., 1993) and mammals (Kalra et al., 1997), implicating NPY in the promotion of earlier sexual maturation (Fraley and Kuenzel, 1993). Furthermore, mutations in NPY favouring heterozygote *Dra*I +/− are associated with an earlier age at first egg (AFE; Dunn et al., 2004), compared to homozygous *Dra*I +/+ or *Dra*I −/− and may be correlated with the total number of eggs laid (Wu et al., 2007). Interestingly, it was determined that these same mutations in NPY *Dra*I influence body weight at the time of sexual maturation, with a higher breeding value determined for the heterozygote (Fatemi et al., 2012), further suggesting a possible link between metabolism and reproduction.

As AgRP is co-expressed with NPY, this peptide likely also influences reproduction in chickens, yet few studies have considered this relationship. It is known that AgRP mRNA levels increase during incubation in hens, however, in this same study, voluntary decrease in feed intake observed in incubating hens was not found to differ from pair-fed hens, while those released from feed restriction demonstrated a significant decline in this peptide (Dunn et al., 2015). Additionally, AgRP mRNA levels in roosters were found to be higher during the photosensitive periods, while declining in periods of photorefractoriness, corresponding to a period with lower body weight and feed intake (Banerjee and Chaturvedi, 2018). AgRP has also been found to play a role in the reproductive system of other species, with mRNA levels declining following ICV of E_2_ in mice (Olofsson et al., 2009), and mRNA levels significantly increasing at the beginning of a breeding season in ewes while lower levels are present at the end of this period (Clarke et al., 2000). Thus, from information gathered from other species, it is likely that in laying hens AgRP expression increases prior to the initial peak of E_2_, at a time when energy is needed for both growth and sexual maturation processes. Levels would then be expected to decrease thereafter, as the hen switches metabolic demand from growth to reproduction. Nonetheless, this hypothesis still needs to be tested.

#### Effects of the Anorexigenic System on Reproduction

There is currently a gap in the literature surrounding the role of POMC during the process of sexual maturation in the laying hen. One study, involving the Shaoxing duck, reported a peak in POMC mRNA levels approximately 30 days prior to the elevation of GnRH-I mRNA (Ni et al., 2011). This is the first reported alteration in POMC expression during this time in an avian species. Throughout a laying cycle, POMC mRNA levels have been reported to remain unchanged among laying and non-laying broiler breeder hens with paired body weight (Dunn et al., 2013), as well as in bantam hens in the laying and incubation phases (Dunn et al., 2015). While this would suggest that this peptide is not implicated in the reproductive status of the hen, the activity of the cleavage products of POMC reveal a contrasting interpretation. Treatment of broiler chicks with α-MSH via ICV showed elevated expression of NPY and AgRP in the IN, indicating a potential homeostatic feedback mechanism to balance the dual control of the melanocortin system (Delp et al., 2017), and an indirect stimulatory role in the control of the HPG axis. Conversely, endogenous opioid peptides have been found to play an opposing role as β-endorphin has been linked to the suppression of LH release and ovulation after administration into the third ventricle of white leghorn laying hens (Sakurai et al., 1986), while [Met]-enkephalin was found to exert an inhibitory response on the release of GnRH in the cockerel by reducing the response to depolarization *in vitro* (Stansfield and Cunningham, 1987) due to the close proximity of these neurons to that of GnRH (De Lanerolle et al., 1981; Sterling and Sharp, 1982). Taken together, these results imply that while the expression of POMC remains constant throughout a laying cycle, evaluation of the cleavage products would be more appropriate to assess the underlying activity of this peptide and its effects on the hypothalamic release of GnRH and or GnIH.

Similar to that of POMC, the role of CART during sexual maturation remains relatively unknown. CART mRNA was initially reported to be primarily present in the hypothalamus and pituitary gland of adult laying hens and its peptide undetectable in the ovary (Cai et al., 2015). Since, it has been established that CART mRNA is present within subsets of follicles with the highest expression in the theca layer of large white follicles compared to the pre-ovulatory follicles (Li et al., 2017), ultimately suggesting that CART does in fact have a role, which has yet to be determined in avian species. When considering mammalian literature, CART has been identified to be under the control of LEP (Douglass, 1995; Gautvik et al., 1996; Kristensen et al., 1998; Rogge et al., 2008), be responsible for regulating the stress response (Koylu et al., 2006), the energy balance (Kristensen et al., 1998; Rogge et al., 2008), and bone remodelling (Elefteriou et al., 2005) in rodents, as well as ovarian follicle development in bovine (Kobayashi et al., 2004; Sen et al., 2007). Specifically in rats, elevated hypothalamic CART mRNA levels were found to decrease GnRH-I interpulse intervals, increasing the frequency (Lebrethon et al., 2000). Additionally, CART mRNA was found to inhibit FSH signaling in cattle (Sen et al., 2007, 2008). Thus, although limited information is available on the role of CART in avian species, as for POMC, literature in mammals suggests an overall suppressive role.

#### Melanocortin Receptors and Reproduction

As previously mentioned, MC5R is the only receptor subtype reported to be expressed in the liver, which is of particular interest as the liver is known to form yolk lipoproteins throughout the laying cycle (Walzem et al., 1999). Since this process is under the influence of E_2_, a potential interaction between the melanocortin and reproductive systems may occur in this organ to control and direct liver metabolism based on the status of the animal. Based on a previous study, MC5R expression appears to be unaffected by E_2_ treatment (Ren et al., 2017). However, although the role of MC5R remains uncertain, recent studies in chicken demonstrated that MRAP is significantly upregulated by E_2_ in the liver (Ren et al., 2017), possibly via peroxisome proliferator-activated receptor gamma (PPAR-γ; Mangelsdorf et al., 1995), as PPAR-γ gene expression increases with rising E_2_ levels and decreases with age (Ren et al., 2017). Interestingly, changes in MRAP were not found to have any influence on MC5R expression in the liver (Ren et al., 2017).

#### Influence of Reproductive Neuropeptides on Appetite Control

While activation of the HPG axis is integrated with a number of hormones, photoreceptors, and signaling pathways, it has become increasingly apparent that these same reproductive neuropeptides have the ability to influence feed intake in avian species, thereby altering the melanocortin system. Just as GnRH and GnIH act as a dual control system on the HPG axis (Bédécarrats, 2015), they have also been observed to play a similar dual control on appetite. For example, in layer chicks GnIH is known to stimulate feed intake (Tachibana et al., 2005) in an orexigenic fashion by stimulating NPY and inhibiting POMC in the hypothalamus (McConn et al., 2014). It has been hypothesized that this effect on feed intake stems from the inhibition of the remainder of the HPG axis, as feed intake has been observed to increase with the decline in steroidogenesis of Japanese quail (Satake et al., 2001), and decrease with E2 administration in laying hens (Jaccoby et al., 1995). While in the laying hen there has been no difference identified in GnIH neuron activity between *ad libitum* fed and feed restricted hens (Ciccone et al., 2007), an increase in GnIH activity is observed after 48 h of deprivation in the Peking duck (Fraley et al., 2013). Whether this effect is species specific or genetically altered by divergent breeding goals requires further investigation. Meanwhile, since GnIH is able to suppress the activity of GnRH-I, it could be hypothesized that this stimulatory neuropeptide would play an anorexigenic role in birds. However, as previously discussed, the endogenous opioid peptide β-endorphin has been reported to have a tonic inhibitory effect on the expression of LH (Sakurai et al., 1986; Stansfield and Cunningham, 1987). Interestingly, processes from CART neurons are in close proximity to GnRH perikarya in numerous mammalian species (Leslie et al., 2001; True et al., 2013) and such a relationship should be evaluated in avian species. If CART does in fact stimulate GnRH-I, evidence of the co-expression of CART with α-MSH, observed in rodents (True et al., 2013), suggests that the activation or suppression of GnRH may depend on the cleavage of the POMC molecule. Further studies will be required to determine whether or not GnRH-I plays an anorexigenic role in the melanocortin system. While this role of GnRH-I is unclear, the activity of the orexigenic response to this neuropeptide has been confirmed with NPY able to stimulate an LH surge in laying hens (Contijoch et al., 1993). However, with the ability of NPY to stimulate corticotrophin releasing factor (CRF) upregulation (Li et al., 2000), and the known integration of CRF neurons with GnRH-I cells observed in rats (Maclusky et al., 1988), it is suggested that this LH surge is likely due to the direct stimulatory effect of NPY on GnRH-I. Altogether, this highlights a possible pathway for the integration of metabolic signals and the HPG axis, leading to the hypothesis that immature hens with higher expression of GnIH would continue to grow due to the stimulation of feed intake. This increase in feed intake will be associated with an elevation in NPY expression, eventually allowing an elevation in GnRH-I to occur to activate the HPG axis.

## Impact of Body Weight and Composition on the HPG-axis

In many species, it has been shown that obesity is strongly linked to reproductive deficiencies. In humans, obesity in women has been linked to poor conception and implantation rates (Brewer and Balen, 2010), while obesity in mice results in a reduction in oocyte and preantral follicle numbers (Sagae et al., 2012). In layers, diet-induced obesity increased proapoptotic effects in granulosa cells through altered steroidogenesis, causing a decrease in reproductive capacity (Walzem and Chen, 2014). However, while obesity is clearly negatively correlated to reproduction, excessively low body weight is also of concern, leading to the development of the “critical weight hypothesis” which was further confirmed in broiler breeders (van der Klein et al., 2018; Zuidhof, 2018). This hypothesis stipulates that puberty in immature animals could not be predicted by age, but rather by the accumulation of body fat stores (Frisch and McArthur, 1974). Over the years, this hypothesis has been put to the test and while insufficient fat stores have been found to delay the onset of sexual maturation in rats, rapid accumulation of these stores can lead to puberty, even if these animals are still well below target body weight (Ronnekleiv et al., 1978), however, the dynamics and pathways behind this concept have yet to be explored in the hen. While studies have considered the effect of food availability on reproductive success in wild species of seasonal breeders, in the domestic laying hen food availability is not a limiting factor and control of the reproductive axis is achieved mostly by modulating environmental conditions, such as photoperiod. As demonstrated in a recent trial involving Lohmann LSL-Lite, current commercial strains do not necessarily require photostimulatory cues in order to initiate the reproductive process (Baxter and Bédécarrats, 2019). This suggests that sexual maturity is not exclusively triggered by photoperiodic cues, but rather hens are required to reach a critical threshold in body weight or fat composition in order to enter lay (Zuidhof, 2018; Baxter and Bédécarrats, 2019). In terms of the HPG axis, this suggests that additional factors can overcome the inhibitory mechanisms in place prior to photostimulation.

This is in line with previous reports showing that a particular body weight target and degree of body fat is required in order to achieve the initiation of maturation in both broiler breeders and quail (Bornstein et al., 1984; Yang et al., 2013), with abdominal fat pad being an accurate indicator of overall fat accumulation in all chickens (Sato et al., 2009). In broiler breeders, hens that had not entered lay prior to 55 woa had a fat pad which was 1.5% of their body weight, while those that entered lay had a fat pad of 2.5%, suggesting that a minimum threshold does exist (van der Klein et al., 2018). Conversely, a study using broiler breeder hens selected for divergent abdominal body fat percentage, referred to as lean and fat hens, demonstrated that lean birds showed an earlier AFE when compared to the fat females, with an overall greater egg production (Zhang et al., 2018), supporting the necessity to avoid excessively exceeding the threshold of body fat percentage of the hen around the time of sexual maturity. Interestingly, these lean and fat hens did not statistically differ in body weight at any time (Zhang et al., 2018). Achieving a critical threshold of body composition during the juvenile stage thus appears required to support the demands for egg formation throughout a laying cycle. Seasonal migratory breeders are believed to possess a sliding body weight set point, referred to as rheostasis, defined as the body weight differences between physiological periods of breeding and migration (Mrosovsky, 1990). Body weight of a common seasonal breeder, the American Kestrel, was maintained at a significantly lower weight throughout the year as long as the hen remains in a non-breeding condition. Interestingly, prior to the breeding period, the non-breeding females in in this study were found to have a lower body weight than those that later entered lay, suggesting the need for an adequate body weight prior to the breeding season to initiate lay. Once they enter a period of breeding, body weight further increased corresponding to the week of and prior to each ovulation, with significant correlations between body weight and concentrations of the sex steroids, estradiol and estrone, attributed to a combination of alterations in fat and protein deposition, along with the maturation of the reproductive tract (Rehder et al., 1986). This suggests various weight set points may be required throughout a reproductive cycle.

**FIGURE 2:**
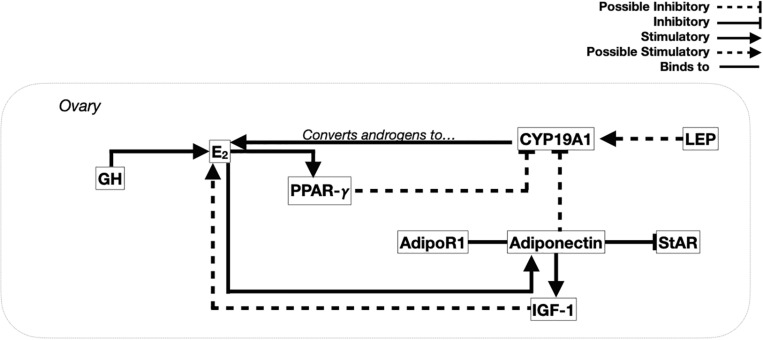
Proposed integration of metabolic factors with maturation of ovarian follicles. In the ovary, growth hormone (GH) binds to the growth hormone receptor first in the theca cell layer of prehierarchal follicles, then in the granulosa cell layer during growth of hierarchical follicles. In prehierarchal follicles, GH stimulates the production of estradiol (E_2_) from the theca layer, further stimulating the early stages of follicular recruitment and development. Once E_2_ is produced, it stimulates the expression of peroxisome proliferated-activated receptor gamma (PPAR-γ), which in turn has the ability to inhibit E_2_ production, forming a negative feedback loop. Prior to activation of the ovary, adiponectin mRNA levels increase in the theca layer allowing binding of adiponectin to its receptor (AdipoR1) present in both cell layers. Adiponectin then down-regulates steroidogenic acute regulatory (StAR) protein and insulin-like growth factor 1 (IGF-1), which are hypothesized to stimulate E_2_ synthesis in the absence of gonadotropins. Leptin (LEP), upon binding to its receptor (LEPR) mostly present in the granulosa layer, may stimulate CYP19A1 to increase E_2_ production while reducing testosterone. Meanwhile, as E_2_ is able to stimulate adiponectin expression in the theca layer and, with adiponectin able to inhibit the production of E_2_, a local negative feedback system between the two has been proposed.

Regulation of body weight and body composition is a very complex process that involves many factors and hormones, some which are also known for their role in the control of reproduction. Although the list is quite exhaustive, for the purpose of this review emphasis will be put on growth hormone (GH) for its involvement in growth and general metabolism, PPAR-γ, adiponectin, and LEP as these hormones and factors specifically control lipid metabolism (Figure 2).

### Peroxisome Proliferator-Activated Receptor Gamma (PPAR-γ)

Peroxisome Proliferator-activated Receptor Gamma is an important regulator of lipogenesis and adipogenesis in mammals (Luquet et al., 2004). PPAR-γ is a member of the nuclear hormone receptor superfamily, binding to the peroxisome proliferator-response element, located in the promotor region of genes directly associated with glucose, and lipid homeostasis (Straus and Glass, 2001). It is highly expressed in all pituitary secretory cells in humans (Bogazzi et al., 2005), as well as adipose tissue of broilers where it plays a major role on fat deposition (Meng et al., 2005; Wang et al., 2008; Zhang et al., 2015). In fact, as this factor is associated specifically with the differentiation of adipocytes and lipid accumulation, it has been linked to NPY which is itself involved in the synthesis of preadipocytes in chicken adipose tissue *in vitro* (Zhang et al., 2015; Shipp et al., 2016). Thus, these studies suggest that PPAR-γ may be a link between the regulation of appetite and body composition. Additionally, elevated expression of this transcription factor has been observed in the liver of broilers selected for fatness in comparison to lean birds (Larkina et al., 2011; Zhang et al., 2015), further demonstrating the ability of this factor to divert nutrients to adipose deposition. Interestingly, variations in PPAR-γ levels have also been associated with genotype, age, and sex (Meng et al., 2005; Sato et al., 2009). In the laying hen, PPAR-γ has been detected in various tissues including the brain, liver and ovary (Sato et al., 2004; Meng et al., 2005; Hojo et al., 2006; Ojano-Dirain et al., 2007; Wang et al., 2012), suggesting a possible role in the control of reproduction. A 23-fold increase in PPAR-γ mRNA was observed in the liver of layers administered high doses of exogenous E_2_ (Lee et al., 2010). This was associated with a corresponding increase in fatty acids, triacylglycerol, and an accumulation of hepatic lipids (Sato et al., 2009; Lee et al., 2010), thus implicating PPAR-γ in the formation of yolk precursors in the liver, an organ under the control of E_2_ (Deeley et al., 1975). If PPAR-γ upregulates the expression of MRAP, as previously discussed, this would suggest that E_2_, through its ability to directly trigger the upregulation of PPAR-γ, has the ability to activate the melanocortin system, diverting energy expenditure from growth to reproduction, linking sex steroids, and energy homeostasis. As a matter of fact, this has also led to the hypothesis that PPAR-γ may play a role in the control of egg production overall, as demonstrated through higher expression levels in high producing laying hens compared to low producing lines (Chen et al., 2010). Beyond egg formation, PPAR-γ has also been suggested to play a role in the control of gonadotropins, with one study hypothesizing that chicken prostaglandin-D synthase protein has the ability to regulate LH-β transcription via PPAR signaling pathways (Chen et al., 2010). Whether this is a direct or indirect effect is not known.

### Adiponectin

Adiponectin is a cytokine predominantly secreted by the adipose tissue with a significant role in lipid and carbohydrate metabolism in mammals (Kadowaki and Yamauchi, 2005). In addition to the breakdown of fatty acids, adiponectin increases insulin sensitivity in mice (Yamauchi et al., 2002), with involvements in energy balance and body weight (Fruebis et al., 2001; Yamauchi et al., 2001). In the chicken, while adiponectin is highly expressed in adipose tissue, it is also expressed in the liver, anterior pituitary, hypothalamus, kidney, skeletal muscle, and ovary (Maddineni et al., 2005; Chabrolle et al., 2007). Plasma concentrations of adiponectin have been shown to decline in broiler chicks between 4 to 8 woa, corresponding to an increase in body weight and a 2-fold increase in abdominal fat pad during this time (Maddineni et al., 2005). While a decline in plasma adiponectin was not observed in birds fasted for 48 h (Hendricks et al., 2009), mRNA levels were found to significantly decline in adipose, liver and anterior pituitary, with other tissues, such as the hypothalamus, remaining unaffected (Maddineni et al., 2005). This implies that while expression may be altered, temporary metabolic changes have little to no influence over the short-term secretion of this hormone. Adiponectin is able to elicit its response through two receptors, AdipoR1 and AdipoR2. While AdipoR1 is primarily found in the skeletal muscle, adipose tissue and diencephalon, AdipoR2 was largely localized to the adipose tissue (Ramachandran et al., 2007), with mRNA and protein of both receptors recently found in theca and granulosa layers of ovarian follicles (Hadley et al., 2020). Signaling pathways are predicted to differ between receptors as AdipoR1 activates AMPK signaling, while AdipoR2 is believed to elicit its response through the transcription factor PPAR-α (Yamauchi et al., 2003). Regarding appetite control, in rodents adiponectin elicited an anorexigenic response through AdipoR1 and its co-localization with the leptin receptor (LEPR) in the hypothalamus, with both receptors present in NPY and POMC neurons (Guillod-Maximin et al., 2009). A similar relationship between AdipoR1 and LEPR should be investigated in avian species to determine the role of adiponectin and leptin in lipid metabolism and overall energy homeostasis within the hypothalamus. Additionally, the possibility of AdipoR1 and AdipoR2 being involved in a permissive, and/or inhibitory role with NPY and POMC should be explored in the hen, as there is the potential for integration with the melanocortin system, as discussed previously in regard to PPAR-γ. Meanwhile, AdipoR1 was upregulated by PRL, while AdipoR2 was downregulated by GH in adipose tissue of mice, (Nilsson et al., 2005), with both hormones inversely associated with adiponectin expression in both mice (Berryman et al., 2004), and humans (Mantzoros et al., 2004). Due to the critical role of these opposing hormones during the reproductive cycle of the hen, further studies should be conducted to determine a possible relationship in chickens. As reported in humans (Yamauchi et al., 2001), plasma adiponectin is negatively correlated to glycaemia in turkey hens (Diot et al., 2015), demonstrating a potential role in elevating insulin sensitivity. In the presence of elevated insulin concentrations, POMC and CART expression has been reported to elevate, while the expression of NPY is inhibited (Porte et al., 2002; Honda et al., 2007; Shiraishi et al., 2008). This is of particular interest as POMC has been found to increase in incubating Silkie hens, a period known to be associated with high PRL levels, compared to their laying counterparts (Sharp et al., 1989; Dawson, 2008; Takeda and Ohkubo, 2019). As AdipoR1 is upregulated in the presence of PRL, it would be expected that adiponectin levels would elevate during this period, yet concentrations have been observed to decline through to the end of production in turkey hens (Diot et al., 2015), requiring further investigation to determine its role throughout a production cycle.

Adiponectin is expressed exclusively in the theca layer of ovarian follicles within both broiler breeder and laying hens, with an autocrine or paracrine effect on steroidogenesis (Chabrolle et al., 2007; Hadley et al., 2020). Interestingly, *in vitro* treatment of porcine granulosa cells with recombinant adiponectin was found to increase steroidogenic acute regulatory protein (StAR) mRNA, along with a reduction in cytochrome P_450_ aromatase, or CYP19A1 (Ledoux et al., 2006). While StAR is responsible for transporting cholesterol across the inner mitochondrial membrane in order to undergo conversion to pregnenolone (Sugawara et al., 1995; Cherradi et al., 1997), CYP19A1 converts testosterone to E_2_. Altogether, this implies that regardless of the ability of adiponectin to stimulate StAR, the decline in CYP19A1 would inhibit the synthesis of E_2_. Recently, granulosa cells from subsets of prehierarchal and preovulatory follicles of broiler breeder and laying hens cultured with recombinant adiponectin reported a decline in StAR mRNA abundance in all follicle groups, with the exception of F4 (Hadley et al., 2020). Additionally, the AMPK signalling pathway has been determined to be activated by adiponectin in the chicken. This AMPK pathway has been shown to differentially regulate StAR depending on the stage of follicular development (Tosca et al., 2006), suggesting that the unequivocal expression of StAR in response to adiponectin *in vitro* can be influenced by a variety of factors and requires further investigation. Furthermore, activation of this pathway led to an increased production of P_4_, along with an elevation in StAR and CYP19A1 levels, in the absence of FSH (Tosca et al., 2006), indicating that another factor may be able to overcome the need for gonadotropin stimulation in avian species. When human recombinant adiponectin was applied to chicken granulosa cells from pre-ovulatory follicles for 36 h in culture, the expression of insulin-like growth factor-1 (IGF-1)-induced P_4_ secretion in preovulatory follicles was upregulated, while LH and FSH-induced P_4_ production in this same subset of follicles decreased (Chabrolle et al., 2007). This further supports the hypothesis that adiponectin is able to influence steroidogenesis within the follicular hierarchy of the chicken ovary, regardless of the status of the HPG axis. Thus, we hypothesize that adiponectin provides a mechanism through which metabolism is able to overcome the need for photostimulation during the activation of the ovary, via the stimulation of IGF-1 induced P_4_ production. Further supporting the link between adiponectin and reproduction, a recent broiler breeder study showed the timing of an E_2_ increase corresponded to the time during which adiponectin declined in the circulating plasma (Grandhaye et al., 2019). However, when sexually immature leghorn chickens were treated with E_2_, adiponectin mRNA abundance was found to be elevated, along with the mRNA levels of AdipoR1, while P_4_ treatment caused a decline in adiponectin mRNA (Hadley et al., 2020). In Huoyan geese, when ovarian granulosa cells were treated with adiponectin, a significant decline was observed in E_2_, while P_4_ concentration increased (Meng et al., 2019). This suggests an overall negative feedback system between adiponectin and the reproductive steroids. Furthermore, plasma adiponectin did not vary during the laying cycle, with a significant decline only found in turkey hens at the end of lay (Diot et al., 2015). Altogether, this indicates that another mechanism is utilized in order to select follicles into the preovulatory hierarchy. As adiponectin contributes to the downregulation of fat deposition (Hendricks et al., 2009; Tahmoorespur et al., 2010), which aligns with the decreased body fat percentage observed in the current commercial broiler breeder (Zuidhof et al., 2014), it is possible that these hens have elevated adiponectin levels, which may be influenced by its high mRNA expression in the liver and anterior pituitary (Maddineni et al., 2005). This would lead to the hypothesis that adiponectin is able to act in the ovary to promote IGF-1 induced P_4_, rather than LH or FSH-induced P_4_. This level of control must be considered to determine the effects of body weight and metabolic status on follicular development and age of first egg.

### Growth Hormone (GH)

Growth hormone is produced primarily by the anterior pituitary gland under the control of hypothalamic growth hormone releasing hormone (GHRH), with additional production in a multitude of other tissues in addition to the hypothalamus (Render et al., 1995). Although initially identified as a purely somatic hormone promoting growth, a reproductive function for GH has been proposed as plasma levels correlate with the onset of lay in pullets (Williams et al., 1992), and the time of ovulation in hens (Harvey et al., 1979b). Injections of GH in immature laying hens increased ovary weight 1 week prior to maturation (Hrabia et al., 2011). However, for this to happen, it is critical that the growth hormone receptor (GH-R) be expressed within the follicles (Lebedeva et al., 2004; Hrabia et al., 2008) at that time. As GH-R expression increases in the ovary around the time of sexual maturation (Hrabia et al., 2008), it can be predicted that a similar GH effect on ovarian follicles would not occur at an earlier age. However, administration of GH increased the number of SWF (Williams et al., 1992), which not only serve as the follicular pool for the remainder of lay, but are also responsible for the production of E_2_ during sexual maturation (Robinson and Etches, 1986). While GH further stimulated the release of E_2_ from the pre-hierarchal follicles (Hrabia et al., 2012), a decline in this steroid hormone occurred within the hierarchy (Hrabia et al., 2014). A relationship between E_2_ and GH can also be seen in the liver, which is responsive to both hormones (Stevens, 2007; Van Anes et al., 2010). GH has been linked to the elevated expression of estrogen receptor beta (ER-β) in the liver (Hrabia, 2015), suggesting an influence of GH on vitellogenesis, as demonstrated in the pigeon (Harvey et al., 1978). Interestingly, white leghorn laying hens with the sex-linked dwarf gene (*dw*) have been found to demonstrate a dysfunction in the GH-R gene, with a missense mutation found in the cDNA (Hull et al., 1993, 1999), reducing the GH-binding activity in the serum and liver without a complete inhibition (Hull et al., 1999). While this mutation does not appear to affect the production rate of heavy type chickens, this dw gene was found to reduce the laying rate of medium and light strains by up to 10% (Guillaume, 1976). Furthermore, these dwarf hens have been determined to be abnormally fat, with a declined ability to mobilize adipose tissue during lay (Guillaume, 1976; Burghelle-Mayeur et al., 1989), suggesting a role for body fat percentage in the ability to maintain high production rates.

At the end of the laying cycle, decreasing GH concentrations have been reported in many avian species (Scanes et al., 1979; Sharp et al., 1979; Bedrak et al., 1981). Thus, it is evident that GH has the ability to control reproduction at the level of the gonads. However, further evidence also supports an effect higher up in the HPG axis as GH-containing neurons are located throughout the hypothalamus of both turkey hens and ring doves, specifically in the PVN, IN, and ME (Ramesh et al., 2000), as well as the MBH in Japanese quail (Berghman et al., 1992), with expression patterns similar to PRL-containing neurons (Ramesh et al., 2000). Thus, similar to the pituitary, GH and PRL dynamics are linked in the hypothalamus. In the pituitary gland of turkey hens during the transition from egg laying to expression of incubation behaviour, PRL cells replace GH cells, as PRL becomes the predominant circulating hormone associated with the cessation of lay (Ramesh et al., 1996). As the hen begins to drop out of production, PRL expression in the avian brain increased (Ramesh et al., 1996), with a corresponding decline in GnRH (Saldanha et al., 1994).

### Leptin (LEP)

After decades of controversy, erroneous publications (Taouis et al., 1998; Ashwell et al., 1999), and hypotheses regarding its existence (Dakovic et al., 2014), the chicken LEP gene was recently discovered embedded in a GC-rich portion of the genome with numerous repeated segments (Seroussi et al., 2016). Unlike in mammals, where LEP is predominantly produced by adipose tissue, gene expression profiles in the chicken show moderate to high LEP mRNA levels in the cerebellum, hypothalamus, cerebrum, adrenal glands, embryonic testis and ovary, along with low levels in adipose tissue, kidney, and heart. Interestingly, expression of its receptor LEPR correlated with 86% of all tissues in which LEP was discovered. This led to the hypothesis that in the chicken, LEP acts mostly in an autocrine, and/or paracrine fashion (Seroussi et al., 2016), rather than the endocrine response observed in mammals (Kershaw and Flier, 2004). This is further supported by the low levels of circulating LEP found in serum (Seroussi et al., 2016). One of the obvious differences between mammalian and avian profiles is the strong gene expression in the pituitary of the hen, while the hypothalamus is the primary site in mammals, suggesting a potential shift in control of homeostasis by this hormone (Dunn et al., 2000; Seroussi et al., 2016). Nonetheless, due to minimal expression detected in adipose and liver tissues in chickens, evidence suggests LEP may not play a role in avian appetite control (Seroussi et al., 2016). Therefore, further investigation is required to determine the stimulatory pathway of this hormone.

As the gene encoding chicken LEP remained elusive despite the characterization of its receptor, most studies conducted in avian species relied on administration of mammalian LEP. LEP signaling was implicated in a number of processes in the domestic chicken, including glucose and insulin activity (Kamohara et al., 1997; German et al., 2009; Huo et al., 2009), with hyperglycemia downregulating LEPR and insulin receptor (INSR; Rancourt et al., 2015). LEP also interacts with the melanocortin system, through POMC/CART and NPY/AgRP neurons (Elias et al., 1998; Davidowa and Plagemann, 2000; Cowley et al., 2001; Nagamori et al., 2003; Takahashi and Cone, 2005). In rats, LEP led to a significant decrease in hypothalamic NPY expression (Schwartz et al., 1996) through the inhibition of NPY and AgRP neurons (Davidowa and Plagemann, 2000; Nagamori et al., 2003; Takahashi and Cone, 2005), while stimulating POMC and CART neurons (Elias et al., 1998; Cowley et al., 2001), thus classifying LEP as an anorexigenic hormone (Balthasar et al., 2004; Williams et al., 2009; Yosefi et al., 2010). However, the anorexigenic effect of LEP may depend on breed or age in chickens, as it had no impact on feed intake in broiler chicks (Sims et al., 2017), although it could increase body weight post-hatch, as well as average daily gain with high doses, while LEP antagonists reversed these effects (Yuan et al., 2017). One rationale for the proposed control of the melanocortin system by LEP is its high level of expression in the brain with undetectable levels in the systemic circulation (Yosefi et al., 2010; Seroussi et al., 2016). In contrast, mammalian LEP is highly expressed in adipose tissue resulting in significant circulating levels, using a short form of LEPR to facilitate its transport across the blood brain barrier (Tartaglia, 1997). In avian species, this short form LEPR is absent (Liu et al., 2007) and LEP exerts its actions by binding to the long form LEPR, activating the JAK-STAT pathway (Adachi et al., 2008; Prokop et al., 2014).

Unfortunately, far less information on a potential role of LEP on the reproductive axis is available in chickens. A study conducted on commercial broiler breeders fed *ad libitum* showed that expression of LEPR was greatest in the granulosa cells of the F3 and F4 follicles, though feed restriction significantly decreased mRNA levels. No differences in relative expression of LEPR within the theca layers of these follicles were reported. Interestingly, LEPR gene expression in the liver was significantly increased in hens fed *ad libitum* compared with feed restricted broiler breeders (Shi et al., 2006). In the laying hen, immunoneutralization of LEP reduced the rate of egg production (Shi et al., 2006), while LEP treatment advanced sexual maturation and ovarian folliculogenesis (Paczoska-Eliasiewicz et al., 2003, 2006). This reported earlier onset of lay was shown to occur through the stimulation of LH, as well as E_2_ and P_4_ production (Paczoska-Eliasiewicz et al., 2006), although a decline in testosterone induced by LEP treatment may result from elevated conversion rate into E_2_ (Sirotkin and Grossmann, 2007). Interestingly, LEP inhibited apoptosis, as indicated by the reduced expression of apoptotic markers within the ovary, along with the promotion of steroidogenesis and differentiation, supporting follicular development (Sirotkin and Grossmann, 2007). Recent studies have also shown that chicken LEP increases the firing rate of neurons within the IN (Bogatyrev et al., 2017), while reducing the expression of the glucocorticoid receptor in the brain (Yuan et al., 2017). This suggests that LEP has the ability to influence signalling within this region of the brain.

## Conclusion

Through divergent breeding goals, selection programs for broiler chickens have largely focussed on rapid offspring growth rate, resulting in poor reproductive efficiency of breeders (van der Klein et al., 2020). In contrast, laying hen selection was primarily based on egg output, with commercial layers more than doubling production over the last 50 years. Through this review, we emphasized that the photoperiodic and metabolic responses utilize many factors that share common pathways and mechanisms to control sexual maturation at all levels of the HPG axis in layer chickens. Although OPN4, OPN5, and VA-Opsin, have all been proposed as deep-brain photoreceptors mediating the photoperiodic response (Foster et al., 1985, 1994; Chaurasia et al., 2005; Halford et al., 2009; Kang et al., 2010; Nakane and Yoshimura, 2010; Davies et al., 2012; Ohuchi et al., 2012), VA-Opsin is the only photoreceptor that has been found to meet all four criteria outlining the activity of the opsin of interest (68,69,71,101; Table 1). In particular, recent evidence of the co-expression of VA-Opsin and GnRH-I strongly suggests this DBP may be a key photoreceptor involved in the photo-induced activation of the HPG axis (García-Fernández et al., 2015).

Beyond photoperiod, it has become clear that sexual maturation can occur prior to photostimulation when a body composition threshold has been met in both layers and broiler breeders (van der Klein et al., 2018; Baxter and Bédécarrats, 2019). Thus, growth is intimately linked to and can influence the initiation of reproduction at the hypothalamic level, and possibly at the level of the ovary. Current knowledge suggests the melanocortin system is strongly associated with the expression of neuropeptides from the HPG axis, with GnIH stimulating the orexigenic peptides AgRP and NPY, while inhibiting the anorexigenic peptides POMC and CART (McConn et al., 2014), as seen in Figure 1. While GnIH downregulates the expression of GnRH, we also hypothesize that elevated expression of AgRP and NPY activate it, thereby allowing the metabolic factors to activate the HPG axis (Contijoch et al., 1993), yet the close proximity of NPY and VIP perikarya in the IN and dorsomedial hypothalamus (DMH) in the redheaded bunting cannot be overlooked (Surbhi et al., 2016). VIP is known to participate in the perception of photoperiod and this close relationship with NPY neurons maybe the key to link metabolism, reproduction and photoperiod (Silver et al., 1988; Bi, 2007; Rastogi et al., 2013; Surbhi et al., 2016) via interaction with GnRH-I and GnIH neurons (Surbhi et al., 2015). Once sexually mature, higher GnRH production results in increased steroidogenesis stimulating POMC and CART expression, thus decreasing feed intake. While CART and α-MSH are hypothesized to positively feedback on GnRH, ACTH stimulates AgRP and NPY (Mountjoy et al., 1994; Kawakami et al., 2000; Strader et al., 2003; Cline et al., 2008; Shipp et al., 2016). However, β-endorphin inhibits LH release (Sakurai et al., 1986; Stansfield and Cunningham, 1987), highlighting the importance of the cleavage of POMC. Interestingly, a recent study reported increased AgRP and NPY mRNA levels with decreased POMC and CART mRNA levels in photosensitive roosters, while the opposite gene expression patterns were observed in photorefractory birds (Stevenson and Ball, 2012).

At the level of the ovary, as shown in Figure 2, additional factors involved in body composition, such as PPAR-γ, GH, and adiponectin, have the ability to stimulate the maturation and maintenance of the hierarchy. With adiponectin increasing IGF-1-stimulated P_4_ production (Chabrolle et al., 2007), it is hypothesized that this hormone can activate the hierarchical development of ovarian follicles prior to activation of the upper levels of the HPG-axis. Interestingly, GH was found to stimulate E_2_ synthesis (Hrabia et al., 2012), which in turn increases the expression of PPAR-γ (Lee et al., 2010). Contrary to mammals, it has been suggested that LEP plays an autocrine/paracrine role at all levels of the reproductive axis in chickens, including the ovary (Seroussi et al., 2016) where it may participate in the maturation of the follicular hierarchy, yet the stimulatory influence on this hormone remains unidentified.

While a number of unanswered questions remain, this review highlights that photostimulation is not the only cue involved in the activation of the reproductive axis in chickens. Metabolic status and/or thresholds can transcend photoperiodic responses. The evidence presented here suggests that although these pathways can act independently, they are in fact synergistic and a coordinated response may optimize reproduction.

## Author Contributions

CH was the primary writer and performed most of the literature search. RR contributed to the section on the reproductive axis as well as the section on metabolic control (specifically the “Additional Factors Involved in the Orexigenic Response with Potential Roles in Reproduction” section). MZ contributed to the metabolism section in particular with insights into practical application and significance. GB participated in the redaction of all sections and supported CH throughout the project as Ph.D. supervisor. All authors contributed to the article and approved the submitted version.

## Conflict of Interest

The authors declare that the research was conducted in the absence of any commercial or financial relationships that could be construed as a potential conflict of interest.

## Abbreviations

α-MSH: Alpha-Melanocyte Stimulating Hormone
β-MSH: Beta-Melanocyte Stimulating Hormone
γ-MSH: Gamma-Melanocyte Stimulating Hormone
ACTH: Adrenocorticotropin Hormone
AdipoR1: Adiponectin Receptor 1
AdipoR2: Adiponectin Receptor 2
AFE: Age of First Egg
AgRP: Agouti-related Peptide
ARC: Arcuate Nucleus
AVT: Arginine Vasotocin
CART: Cocaine and amphetamine-regulated transcript
CORT: Corticosteroid
CYP19A1: Cytochrome P_450_ aromatase
DA: Dopamine
DBP: Deep-Brain Photoreceptor
DIO2: Type 2 Deiodinase enzyme
DIO3: Type 3 Deiodinase
E_2_: Estradiol
ER- β: Estrogen Receptor Beta
FSH: Follicle-Stimulating Hormone
GH: Growth Hormone
GH-R: Growth Hormone Receptor
GHRH: Growth Hormone-releasing hormone
GnIH: Gonadotropin-Inhibiting Hormone
GnIH-R: Gonadotropin-Inhibiting Hormone Receptor
GnRH-I: Gonadotropin-Releasing Hormone I
GnRH-II: Gonadotropin-Releasing Hormone II
GnRH-RI: Gonadotropin-Releasing Hormone Receptor I
GnRH-RIII: Gonadotropin-Releasing Hormone Receptor III
HPA axis: Hypothalamic-Pituitary Adrenal axis
HPG Axis: Hypothalamic-Pituitary Gonadal Axis
IGF-1: Insulin-like Growth Factor 1
IN: Infundibular Nucleus
INSR: Insulin Receptor
LD: Long Day
LEP: Leptin
LEPR: Leptin Receptor
LH: Luteinizing Hormone
LSO: Lateral Septal Region
MBH: Medial-Basal Hypothalamus
MC1R: Melanocortin Receptor subtype 1
MC3R: Melanocortin Receptor subtype 3
MC4R: Melanocortin Receptor subtype 4
MC5R: Melanocortin Receptor subtype 5
ME: Median Eminence
MEL: Melatonin
MRAP: Melanocortin Receptor Accessory Proteins
NPY: Neuropeptide Y
OPN1: Opsin 1
OPN3: Opsin 3
OPN4: Melanopsin (Opsin 4)
OPN5: Neuropsin (Opsin 5)
P_4_: Progesterone
PMM: Pre-mammillary Nucleus
POMC: Pro-opiomelanocortin
PPAR- γ: Peroxisome Proliferator-activated Receptor Gamma
PRL: Prolactin
PT: Pars Tuberalis
PVN: Paraventricular Nucleus
PVO: Paraventricular Organ
RGR: Retinal G-coupled Receptors
SD: Short Day
StAR: Steroidogenic Acute Regulatory Protein
T_3_: Triiodothyronine
T_4_: Thyroxine
TMTs: Teleost Multiple Tissue Opsins
TSH: Thyroid-Stimulating Hormone
VA-Opsin: Vertebrate-Ancient Opsin
VIP: Vasoactive Intestinal Peptide.
